# Porosity of Bleb Capsule declines rapidly with Fluid Challenge

**DOI:** 10.5005/jp-journals-10008-1208

**Published:** 2016-10-29

**Authors:** Surinder S Pandav, Craig M Ross, Faisal Thattaruthody, Ritambhra Nada, Nirbhai Singh, Natasha Gautam, Stephen Beirne, Gordon G Wallace, Mark B Sherwood, Jonathan G Crowston, Michael Coote

**Affiliations:** 1 Professor, Advanced Eye Center, Postgraduate Institute of Medical Education and Research, Chandigarh, India; 2 Research Fellow, Center for Eye Research Australia, University of Melbourne Melbourne, Victoria, Australia; 3 Senior Registrar, Advanced Eye Center, Postgraduate Institute of Medical Education and Research, Chandigarh, India; 4 Professor, Department of Pathology, Postgraduate Institute of Medical Education and Research, Chandigarh, India; 5 Assistant Professor, Advanced Eye Center, Postgraduate Institute of Medical Education and Research, Chandigarh, India; 6 Senior Registrar, Advanced Eye Center, Postgraduate Institute of Medical Education and Research, Chandigarh, India; 7 Senior Research Fellow, Intelligent Polymer Research Institute/AIIM Faculty, University of Wollongong, Wollongong, New South Wales, Australia; 8 Professor, Intelligent Polymer Research Institute, ARC Centre of Excellence for Electromaterials Science, University of Wollongong Wollongong, New South Wales, Australia; 9 Professor, Department of Ophthalmology, University of Florida, Gainesville Florida, United States; 10 Managing Director, Center for Eye Research Australia, University of Melbourne Melbourne, Victoria, Australia; 11 Associate Professor, Center for Eye Research Australia, University of Melbourne Melbourne, Victoria, Australia

**Keywords:** 3D Printed implant, Aqueous outflow, Capsular porosity, Filtering surgery, Glaucoma drainage device, Glaucoma, Hydraulic conductivity, Rabbit model, Rapid failure.

## Abstract

**Introduction:**

The porosity of the fibrous capsule around a glaucoma drainage device (GDD) may be the most important functional attribute. The factors that determine capsular porosity are not well understood. Failed GDD surgeries are usually associated with thick impervious capsules and components of aqueous have been implicated in this process.

**Purpose:**

In this study, we interrogated the effect of passage of nonaqueous fluid on capsular porosity in mature (but aqueous naïve) blebs in a previously reported GDD model (the “Center for Eye Research Australia Implant”).

**Materials and methods:**

The study was performed at two centers using 17 New Zealand White (NZW) rabbits. An experimental GDD was implanted into the subconjunctival space but without connection to the anterior chamber. After 28 days, balanced salt solution (BSS) was passed through the implant for 30 to 40 minutes at 12 mm Hg. Capsular porosity was measured as flow (μL/min) at a constant pressure. Porosity of the capsule was retested at 3 and 6 days.

**Results:**

There was a marked reduction in capsular porosity within 3 days of exposure to BSS (fluid challenge). Even though the baseline porosity was significantly different in the two groups (3.00 ± 0.5 μL/min and 29.67 ± 12.12 μL/min, p < 0.001), the effect of passage of BSS was similar. Capsular porosity fell by approximately 80% in both groups from baseline after single BSS challenge. Capsular thickness was significantly less in Advanced Eye Center (AEC) rabbits at baseline. There was no change in the capsular thickness before and after single fluid challenge.

**Conclusion:**

Passage of BSS at physiological pressures for under 40 minutes caused marked reduction in the porosity of the fibrous capsule within 3 days. This was not associated with any significant thickening of the fibrous capsule within this time frame.

**How to cite this article:**

Pandav SS, Ross CM, Thattaruthody F, Nada R, Singh N, Gautam N, Beirne S, Wallace GG, Sherwood MB, Crowston JG, Coote M. Porosity of Bleb Capsule declines rapidly with Fluid Challenge. J Curr Glaucoma Pract 2016;10(3):91-96.

## INTRODUCTION

Glaucoma surgery has relatively high rates of complications and failures. Failure rates vary depending upon the criteria used; for studies using the most stringent criteria over half of trabeculectomies have failed at 5 years from operation,^1,2^ though many other studies report outcomes better than this. Failure of glaucoma surgery is usually defined by postoperative intraocular pressure (IOP) measurements exceeding target, although the target has been variously defined in published literature.^3^ Intraocular pressure in the unoperated eye is determined by outflow facility - a combination of trabecular and nontrabecular outflow. Following glaucoma surgery the IOP is principally determined by the surgically created outflow.

Previous work has shown that the key determinant of surgical outflow is bleb capsule porosity.^4^ Failure of glaucoma surgery to produce target IOP is most often due to failure of the bleb capsule to retain sufficient porosity to allow the necessary outflow.

Glaucoma drainage devices (GDDs) have gained in popularity in recent years but like trabeculectomy, they also have high failure rates.^5,6^ Existing drainage devices are frequently associated with the formation of a thick fibrous capsule over the implant.^7,8^ Implant size, shape, material, aqueous effects on tissue, and inherent tissue response to surgery all have been reported to influence the amount and nature of the fibrous capsule forming around the device.^9,10^

We have previously reported on a new model of interrogating glaucoma surgery (the “Center for Eye Research Australia (CERA)” implant). Using this model, porosity of the bleb capsule has been studied in the New Zealand White (NZW) rabbit model.^11,12^ In short, all NZW rabbits had thick capsules with very low porosity at 4 weeks when the implant had been connected to the anterior chamber and the plate had been bathed in aqueous. Histologically, bleb capsules have been reported to have marked subconjunctival/bleb capsule condensation of collagen which is (relatively) impervious.^13,14^ Collagen fibrils along with deposition of glycosaminoglycans and proteoglycan core proteins are associated with low tissue hydraulic conductivity. Proteoglycan complex amplified by collagen fibril network has been reported to play a major role in determining hydraulic conductivity.^15^

Although changes to the capsule can be observed occurring in parallel to the loss of porosity, the processes leading to reduction in porosity are not well understood. Biologically active molecules in the aqueous have been implicated, but there may be other mechanisms contributing toward failure of glaucoma surgery.

In this study, we explored the effect of flow of nonaqueous fluid [balanced salt solution (BSS)] around the implant. The study was designed to exclude the effect of aqueous on capsular porosity and minimize the impact of inflammation and foreign body reaction associated with drainage device surgery.

## MATERIALS AND METHODS

Initial study (n = 7) was conducted at CERA, Melbourne, Australia (group I). A second series of experiments (n = 10) was done at the Advanced Eye Center (AEC), Postgraduate Institute of Medical Education and Research, Chandigarh, India (group II). In addition, five rabbits (group III) that did not undergo porosity measurements were used as histology controls (28 day baseline) at AEC. At both centers, the same investigator (SSP) conducted the surgical and measurement procedures and the same implants were inserted. The same equipment was used to make porosity measurements at both the places. Both experiments were performed on NZW rabbits, although animal stocks in each place differed in terms of lineage and conditions. Both sets of animals were kept in the approved animal holding facilities under conditions complying with the Association for Research in Vision and Ophthalmology (ARVO) statement for the use of animals in ophthalmic research. Institutional ethics committees at both the institutions approved the study, separately.

### Surgical Procedure

Details of the procedures have been reported earlier.^11,12^ Briefly, an experimental glaucoma drainage device (“CERA GDD”) was implanted in one eye under general anesthesia. The right eye had a limbal conjunctival incision made in the superotemporal quadrant, and the implant was placed and secured there. In this experiment, neither of the tubes was placed in the anterior chamber. Instead, at least 10 mm of both tubes was left plugged and then placed under the conjunctiva, in the adjacent superior quadrant, which was closed with 8-0 Vicryl sutures. Antibiotic ointment and corticosteroid drops were applied at the end of the procedure. Antibiotic eye drops were continued for 3 days in bid dosage, whereas corticosteroid drops were discontinued after 24 hours.

### Porosity Measurements

**Fig. 1:**
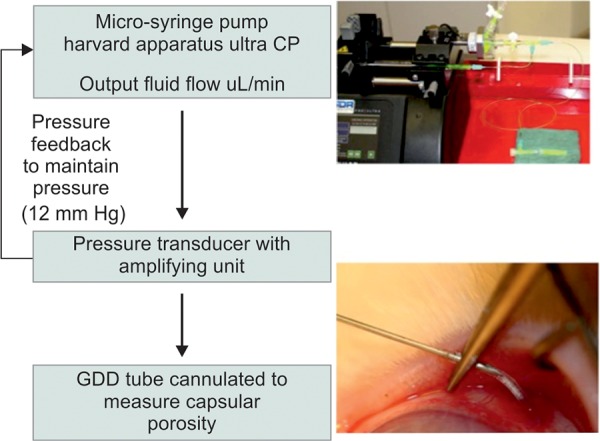
Experimental setup for measuring capsular porosity

Twenty-eight days postinitial tube surgery, the animals were anesthetized and the implant was tested for capsule porosity. To measure outflow, one of the tubes was accessed via a conjunctival incision under sterile conditions, cannulated, and then connected to a pressure transducer-controlled syringe pump (PHD Ultra CP 4400, Harvard Apparatus). The other tube remained blocked. The flow of BSS into implant capsules was measured at 12 mm Hg pressure for 5 minutes after stabilizing the system. After obtaining the porosity values, the fluid was allowed to pass through the capsule freely for 30 minutes (fluid challenge) maintaining a pressure of 12 mm Hg. The implant tube was then occluded and tucked back under the conjunctiva for subsequent measurements (Fig. 1).

During the procedure, the tube was exposed and cannulated over 10 mm away from the plate. The process of testing was minimally disturbing to the conjunctiva, with no inflammation noted over the plate during or after the testing procedure. No steroids were used in the postoperative period.

Three days after the initial measurement (fluid challenge), the capsular porosity was measured again using the same procedure. In some animals, we also measured porosity a 3rd time 3 days after the 2nd measurement, i.e., 6 days after the initial fluid challenge. After the final measurement, the animal was euthanized and eyes were enucleated for histology and preserved in 10% formaldehyde solution.

### Statistical Methods

Differences in the porosity of the fibrous capsules at different time points, at baseline, 3 days later, and 6 days postinitial cannulation were tested for significance using t-test for paired samples. For comparisons between data from two centers, t-test for unpaired samples was used.

## RESULTS

Reliable results could be obtained from seven animals in group I and nine in group II. Observations at various time points where a consistent reading could not be obtained due to a leak or block in the system were excluded from the data analysis.

### Group I Data

Mean capsular porosity in an aqueous naïve capsule prior to challenge was 3.0 ± 0.79 [Standard Error of Mean, (SEM)] μL/min. After a single fluid challenge, it reduced significantly to 0.55 ± 0.10 (SEM) μL/min (p = 0.028) when measured 3 days later. A 3rd measurement was performed on only 2 animals and showed no change in porosity from the 2nd reading, and this was not included in analysis because of the low number.

### Group II Data

The mean capsular porosity was 28.94 μL/min ± 4.28 (SEM) at 4 weeks in an aqueous naïve capsule. This is substantially higher than that in group I, although it was consistent throughout this animal cohort. In response to fluid challenge, the porosity fell significantly to 7.08 ± 2.41 (SEM) μL/min 3 days after the first measurement (fluid challenge) (p < 0.001). All animals in group II had a 3rd measurement and no further reduction in porosity was observed when tested at day 6 (after two fluid challenges). Porosity at day 6 was 7.0 ± 2.88 (SEM) μL/min, which was not significantly different from day 3 porosity (p = 0.659).

### Combined Data

Group I animals had a 75 ± 22% [mean ± standard deviation (SD)] reduction in the porosity after one fluid challenge. Group II animals had 80.76 ± 14.60% (SD) reduction in the porosity of the fibrous capsule after one fluid challenge. Details are presented in Tables 1 and 2. Porosity values at all time points were significantly different between the AEC and CERA rabbits, perhaps reflecting the different lineage and breeding conditions of the animals. In spite of this, the magnitude of effect of fluid challenge on capsule porosity was comparable (p = 0.752) in the two groups.

### Histology

Histology was studied at the following time points. Baseline control at 4 weeks (no exposure to aqueous or BSS (n = 5, group III), after 3 days of first fluid challenge (n = 5), and after 6 days of first challenge. All eyes were stored in 10% formaldehyde until the time of processing at room temperature. Histology sections were stained with hematoxylin and eosin (H&E) stain and examined under magnification to identify and measure capsular thickness. The fibrous capsular thickness at baseline control in AEC rabbits was 40.13 ± 6.48 μm (mean ± SD), which was significantly thinner to the no-flow CERA rabbits at 4 weeks (52.35 ± 2.74 μm (p = 0.017, t-test). Capsular thickness in AEC rabbits at no-flow baseline (40.13 ± 6.48) was not significantly different from capsular thickness 3 days after the fluid challenge (43.79 ± 7.72) (p = 0.347, t-test).

**Table 1:** **Table 1:** Porosity of the fibrous capsule in AEC rabbits before and after the fluid challenges

*Rabbit*		*Baseline*		*3 days*		*6 days*	
1		11.22		1.42			
2		19.40		0.27		0.36	
4		33.95		13.59		22.43	
5		34.01				9.92	
6		46.46		17.28		4.95	
7		15.72		3.05		3.93	
8		37.29		8.12		7.98	
9		33.51		5.82		0.40	
10		35.51		8.26		7.34	
Mean		29.67		7.23*		7.16**	
SD		12.12		6.37		7.62	
SEM		4.04		2.25		2.69	
n		9		8		8	

Microliters/min at 12 mm Hg pressure; n = No. of valid observations; *p < 0.001; and **p=0.752 (between day 3 and day 6)

**Table 2:** **Table 2:** Porosity of the fibrous capsule in CERA rabbits before and after a fluid challenge

*Rabbit*		*Baseline*		*3 days*	
1		0.69		0.55	
2		1.36		0.65	
3		1.71		0.85	
4		1.89		0.26	
5		3.95		0.32	
6		5.5		0.36	
7		5.91			
Mean		3.00		0.50*	
SD		2.10		0.23	
SEM		0.79		0.09	

Microliters/min at 12 mm Hg pressure; and *p = 0.028

**Graph 1:**
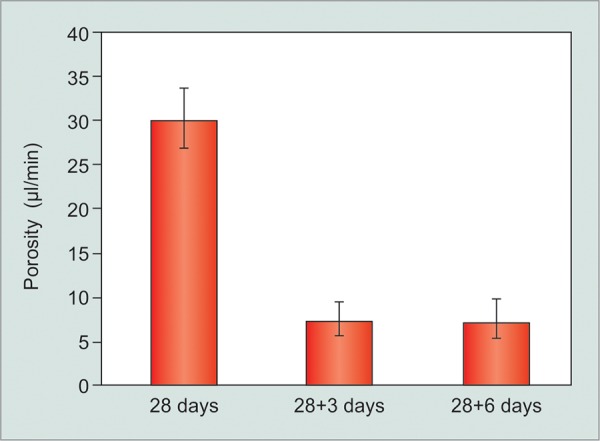
Reduction in capsular porosity (μL/min) following fluid challenge in AEC rabbits

**Graph 2:**
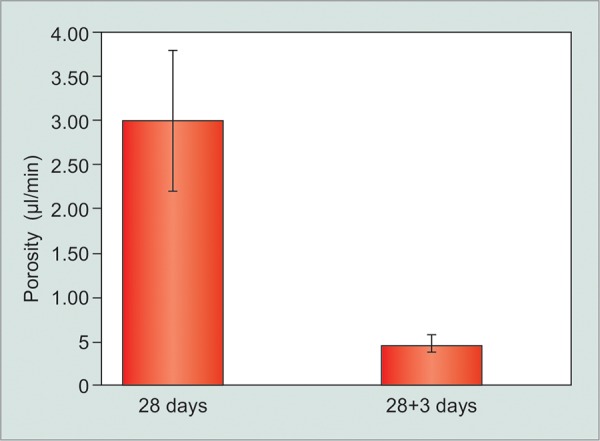
Reduction in capsular porosity (μL/min) following fluid challenge in CERA rabbits

## DISCUSSION

We implanted CERA GDD described elsewhere in two different strains of NZW rabbits.^11,12^ The GDD was left *in situ* for 28 (±2) days without exposure to aqueous. By testing the implants at 28 days, the initial inflammatory process is likely to have resolved and the effect of “fluid stress” is thus (mostly) isolated from the surgical trauma and foreign body reaction. After a brief exposure (~40 min at 12 mm Hg) to BSS, the capsular porosity of the implant capsule decreased by 75 to 80% over the following 72 hours (Graphs 1 and 2). This rapid reduction in function of a GDD has not been reported previously. The measurement system to record porosity employed in this study has been developed by us and been shown to be reliable and repeatable.^12^

There are a number of factors that are argued to influence the functioning of a GDD: These include the material, design, shape, and size of the GDD plate. However, in a systematic review Hong et al^16^ compared five commonly used GDDs and found that most devices lowered IOP between 72 and 79% and IOP lowering was not significantly different among them. It may be inferred from this that the magnitude of effect on outflow of plate material and design is relatively insignificant.

**Graph 3:**
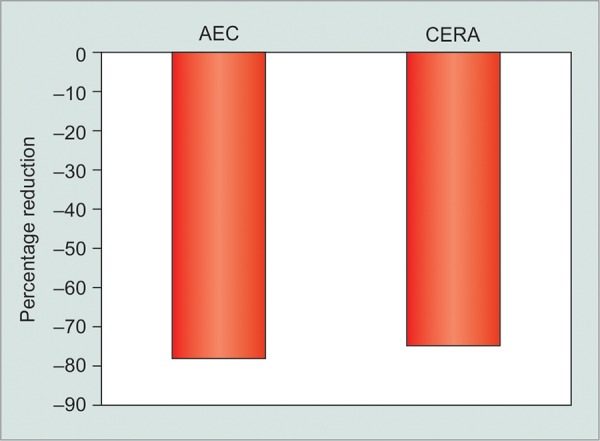
Percent change in capsular porosity in AEC and CERA rabbits following a single fluid challenge

In our study, we found significant difference in the porosity values in AEC and CERA rabbits. The AEC group was significantly more porous than the CERA group (p < 0.001, unpaired t-test) at both 28 days and 28+3 days time points. Both the groups received the same GDD using the same techniques of implantation and porosity measurements in the same animal species. Therefore, this difference is not because of device characteristics but because of differences in response of the tissue to the implant. Different strains of the same species of animals are known to mount different tissue reaction in response to injury.^17^ In our study, the different porosity values were likely to be due to difference in the strain and environment of the NZW rabbits. What was remarkable, however, was that response to fluid challenge was similar in both the groups (Graph 3). Single fluid challenge of 30 to 40 minutes resulted in 75 and 80% reduction in the porosity values within 3 days of exposure, in CERA and AEC rabbits respectively. Thus, despite differences in the capsular porosity of the two different rabbit populations at 1 month postimplantation, the effect of fluid challenge was very similar (Graph 3).

**Figs 2A to D:**
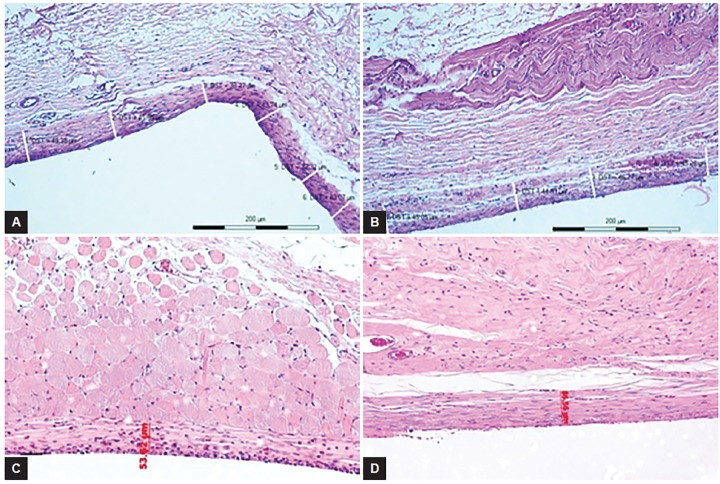
Thickness of the fibrous capsule on histology (H&E 10×): (A) AEC rabbit 28 days after implantation not exposed to fluid; (B) AEC rabbit 28 + 3 days after implantation following single fluid challenge; (C) CERA rabbit 28 days after implantation not exposed to fluid; and (D) CERA rabbit 28 + 6 days after implantation following a single fluid challenge

Aqueous humor has been implicated in failure of the filtering surgery due to presence of certain factors that stimulate fibrosis in the subconjunctival space.^18^ Aqueous humor from glaucoma eyes has been reported to support growth of subconjunctival fibroblasts in tissue culture^19^ and has been found to have increased levels of TGF-β2.^20^ In our study, reduction in capsular porosity was seen after exposure to BSS, demonstrating that there are reasons other than aqueous cytokines, for capsule porosity to fail. In this study, we did not investigate the molecular mechanisms responsible for reduction in capsular porosity. However, we did measure capsular thickness in histological sections to identify changes in capsule dimensions associated with decrease in porosity. In neither group was there a demonstrable increase in capsule thickness at the experimental time points (Figs 2A to D).

Measurement of the dimensions of the identifiable histologic capsule is an intrinsically coarse quantification: The dimensions depending upon the site of the section, the angle and mechanism of sectioning. Even with these caveats, we feel it is important to note that decrease in cap-sular porosity occurred without demonstrable changes to the thickness of the capsular wall. Thickening of the capsule occurs later and may be a primary or a secondary event - secondary to increased pressure (hydrostatic stress) in the capsule. The capsules in the AEC rabbits were significantly thinner and more porous than the CERA rabbits at baseline. There is likely to be differences in the tissue response to the surgical trauma in the two different strains of the same species.^17^ Despite differences in the capsular porosities in two strains of rabbits, the effect of fluid challenge in the two groups was almost identical, giving credence to the hypothesis above.

Wound healing is a complex process, which is characterized by a number of cellular and extracellular events. Cellular components are predominantly inflammatory cells, which transform to fibroblasts to produce various extracellular components including collagen. Activated fibroblasts, in response to injury, proliferate and migrate to the wound site and play a very important role in tissue repair and remodeling.^21^ Wound healing is regulated by a number of cytokines and growth factors. In rabbit eyes, wounds heal rapidly leading to scar formation by 4 weeks. In our experiments, we let the wounds heal for 4 weeks, before giving fluid challenge, so as to isolate the effect of fluid challenge from that of wound healing. Rapid reduction in capsular porosity indicates that there may be other mechanisms, not directly related to wound healing, responsible for failure of glaucoma surgery. Further studies would be needed to explore these mechanisms.

In summary, a brief exposure to fluid reduces the porosity of the fibrous capsule, of an experimental GDD, markedly and rapidly. Significantly, this happens when the effect of surgical trauma, inflammation, and wound healing process is more or less over and also in the absence of any “aqueous cytokines,” thus pointing toward other mechanisms, which needs further study.
